# Maternal employment characteristics as a structural social determinant of breastfeeding after return to work in the European Region: a scoping review

**DOI:** 10.1186/s13006-024-00643-y

**Published:** 2024-05-28

**Authors:** Pauline Brugaillères, Séverine Deguen, Sandrine Lioret, Sahar Haidar, Corinne Delamaire, Emilie Counil, Stéphanie Vandentorren

**Affiliations:** 1 Bordeaux Population Health Research Center, U1219, Inserm, University of Bordeaux, 146 Rue Léo Saignat, Bordeaux, France; 2 Center for Research in Epidemiology and StatisticS (CRESS), Université Paris Cité and Université Sorbonne Paris Nord, Inserm, INRAE, Paris, France; 3 Santé Publique France, Saint-Maurice, France; 4 Institut National d’études Démographiques (INED), Aubervilliers, France; 5 Institute of Interdisciplinary Research On Social Issues (IRIS), UMR8156 CNRS, EHESS, U997 Inserm, SPN, Aubervilliers, France

**Keywords:** Working mother, Infant and young child feeding practice, Social determinants of health

## Abstract

**Background:**

The European Region has the lowest rate of exclusive breastfeeding at 6 months worldwide. Improving work-related breastfeeding issues is important given that women may have difficulties combining work and breastfeeding, especially those in precarious working situations, which adds to their adversity. This scoping review overviews research on the maternal employment characteristics that support breastfeeding continuation after return to work in the European Region.

**Methods:**

Studies published from 2013 to 2023 were collected from Scopus, PubMed, and PsycInfo. Quantitative and qualitative studies published in English or French that explored the association between maternal employment characteristics and any breastfeeding status, duration, or experience were included. Participants included were mothers of healthy children who continued breastfeeding after resuming work. The main determinants were work-related factors that can lead to socially differentiated working conditions, including type of employment (e.g., occupation, employed/self-employed status, type of contract, working time, occupational prestige), working conditions (e.g., work schedule, decision latitude, latitude to organize worktime), and work environment (e.g., occupational exposure, family-friendly workplace policy, social support). The geographic area encompassed countries included in the World Health Organization European Region.

**Results:**

Of the 693 single studies retrieved and screened, 13 were included in the review. Eight studies focused on combining work and breastfeeding, while the others had a broader spectrum by investigating breastfeeding determinants. The represented countries were Spain (*n* = 4), France (*n* = 4), UK (*n* = 2), Ireland (*n* = 2), and the Netherlands (*n* = 1). Results highlighted the heterogeneity of measures, time frames, and fields of inquiry, thus revealing a lack of conceptual framework regarding the links between work, breastfeeding, and social health inequalities. Nonetheless, being self-employed, working in a non-manual profession with time flexibility, having lactation rooms at work, being supported by co-workers, and having a breastfeeding workplace policy were salient factors that supported breastfeeding in working mothers.

**Conclusions:**

Supporting working mothers who choose to breastfeed is important given the myriad of adverse factors faced by mothers and their children. These results advocate for targeted actions at the workplace such as time flexibility, breastfeeding facilities, and the promotion of breastfeeding-friendly policies.

**Supplementary Information:**

The online version contains supplementary material available at 10.1186/s13006-024-00643-y.

## Background

Breastfeeding rates remain relatively low in high-income countries, particularly in the WHO European Region, which has the lowest rates of exclusive breastfeeding in infants aged 6 months compared with other regions, standing at about 25% [1]. Breastfeeding practices vary substantially across high-income countries and within the European Region [2]. As revealed by a survey comparing data from 11 European countries, between 56% (Ireland) and 98% (Norway) of infants were reported to receive any human milk after birth; at 6 months, 38% (Italy) to 71% (Norway) of infants were continuing breastfed, while 13% (Denmark) to 39% (Netherlands) were exclusively breastfed [3]. These cross-national variations in breastfeeding practices may be partially explained by the various social policies in place. Maternity leave regulations differ substantially across the European Region: countries like Sweden, Finland, and Portugal, which offer lengthy and well-compensated maternity leave and have greater uptake, flexibility, and division of leave between parents, show better breastfeeding outcomes in terms of initiation and duration [4].

Indeed, policy attributes are one of the five types of determinants for successful breastfeeding, together with community, health care-related, psycho-social, and sociodemographic attributes [4]. According to the conceptual model proposed by the 2016 Lancet Breastfeeding Series, breastfeeding determinants operate from the most distal levels – i.e., sociocultural context, formula milk industry, health system, family or community, and workplace or employment – to the most proximal levels – i.e., individual factors such as mother and infant attributes and mother-infant relationship [5]. From a socioecological perspective, regulations play the most crucial role in breastfeeding initiation and duration rates such as the existence of baby-friendly hospitals, the international code of marketing for breast-milk substitutes, and maternal, paternal, and parental leave [5, 6]. In the workplace, employers have legal obligations toward lactating mothers, although public policies are still needed for working women to effectively support their choice to breastfeed. Moreover, employment is sometimes conceptualized as the relationship between a woman’s productive and reproductive work; because breastfeeding is sex-specific, it challenges the feminist principle of gender-neutral child rearing [7]. Indeed, the socioecological framework does not take into account how gender is inherently connected with breastfeeding at the structural, cultural, and personal levels such as the place of motherhood in women’s lives, the sexualization versus maternal function of their bodies, and the issue of personal choice [4, 8].

Returning to work while still breastfeeding remains the main challenge faced by lactating mothers [5, 9, 10]. Work-related factors include working full-time, not having access to a suitable place to express and store breast milk, not being supported by co-workers, and returning to work earlier, which all impair breastfeeding intention and practices, including initiation and duration [5]. Removing work-related breastfeeding barriers is especially important given women’s active participation in the labor force. Furthermore, it has been shown that supporting breastfeeding reduces sick leave due to child illness [11]. In contemporary Western societies, even though breastfeeding is praised particularly for its health benefits, there is considerable cultural stigmatization around the current practice of breastfeeding [12], and women may face many difficulties when trying to combine work and breastfeeding. This is especially true for women experiencing socioeconomic disadvantage. Indeed, women with low education level are frequently in low-skilled or precarious employment, characterized by non-supportive breastfeeding environments (e.g., manual labor, full-time, lack of flexibility) [13, 14].

The macro-theoretical framework proposed by the WHO Commission on Social Determinants of Health gives some insight into the relations between employment and health inequalities [15] (Additional file 1). From this, and with the aim of better supporting working women who choose to breastfeed, the present study proposes a deeper understanding of the work-related factors that may hinder this personal/family choice and that may, in turn, worsen social inequalities in maternal and child health. To our knowledge, no study to date has reviewed the structural social determinants of breastfeeding in Europe such as maternal employment in light of the social inequalities in breastfeeding practices after return to work. To fill this gap, the present scoping review aims to identify the maternal employment characteristics that support any breastfeeding continuation after resuming work in the European Region.

## Methods

This scoping review was guided by the Joanna Briggs Institute’s approach to scoping reviews [16] and compliant with the PRISMA-ScR checklist [17].

### Inclusion criteria

Full-text peer-reviewed articles using quantitative and/or qualitative methods and published in English or French between 2013 and 2023 were included according to the following inclusion criteria: (1) Population: mothers of a healthy child with an experience of breastfeeding after resuming work; (2) Outcomes: any breastfeeding duration (i.e., exclusive, predominant, or partial), breastfeeding status, or breastfeeding experience after returning to work; (3) Main determinants: any maternal employment factors that can lead to socially differentiated working conditions, including organizational aspects such as work type, work schedule, worktime flexibility, or type of contract as well as environmental factors like occupational exposure, arduousness, or social support at work; (4) Geographic coverage of the study: countries in the WHO European Region.

### Exclusion criteria

Articles based on interventional studies, opinion pieces, editorials, case studies, or any types of reviews were excluded. Since we focused on mothers choosing to combine breastfeeding and work, studies that only reported associations between breastfeeding practices and maternity leave duration or return to work timeframe were excluded. For the same reason, we also excluded studies focusing solely on breastfeeding intention or initiation, which are events that occur upstream of the return to work. Finally, we excluded studies that only investigated employment as a dichotomous variable (i.e., working vs not working).

### Search strategy

Three electronic databases were used, including Scopus, PubMed, and PsycInfo for relevant articles published in the past 10 years (database searches were conducted on October 22, 2022, and updated on March 20, 2023). The search strategy was first developed in Scopus using proximity operators (e.g., W/3 means that two keywords of interest must be within a maximum distance of three words) and was as follows: (TITLE (Breastf* OR "Breast F*" OR (mother* W/3 milk) OR "Infant Feeding") AND (TITLE-ABS-KEY(((*employ* OR work* OR occupation* OR Job) W/3 (mother OR maternal OR women)) OR "work related" OR "Occupation* related" OR Workplace OR ((parental OR matern* OR Mother OR Breastf* OR "Breast F*") W/3 leave) OR ((job OR Work* OR *employ* OR Occupation*) W/3 (characteristic OR Status OR condition OR Schedule)) OR Shift-work* OR Shiftwork OR "return* to work" OR self-employed) OR KEY("Women Working"))). This search was then adapted to each of the different databases (Additional file 2).

### Screening

After eliminating duplicates, P.B. screened all titles and abstracts using a priori eligibility criteria (e.g., type of paper, country, targeted population, breastfeeding outcome). Then P.B. read the full-text articles of the remaining references to confirm their eligibility; a double-check was carried out at 20% by S.D. (*n* = 17/87; 89% agreement), with any conflicts being resolved by a third reviewer (S.V.).

### Data synthesis and analysis

For each study, data were extracted and summarized in several tables. The following information was reported:

- General information concerning the author’s name, country, and study date;
- Main study characteristics: study design, period, location, statistical methods, and population size;
- Participant characteristics including information on confounders;
- Work-related factors considered to support (or not) breastfeeding when returning to work;
- Outcome definitions including any, exclusive, or predominant breastfeeding;
- Main findings concerning assessments of association, including odds ratios (ORs), hazard ratios (HRs), relative risks (RRs), and other metrics measuring the strength of association of maternal employment characteristics with breastfeeding duration, employment status after returning to work, and experience of breastfeeding as reported in qualitative studies (e.g., work-related barriers and facilitators).

When several measures of association were available for a given outcome, we reported those from the fully adjusted models.

### Description of maternal work-related variables

We grouped the work-related variables into three main dimensions described as follows:

- **Type of employment** refers to the terms that govern the organization of work, generally stated in the contract between the employer and employee, and includes the occupation, work status (employed/self-employed), type of contract (permanent/fixed-term/temporary), working time (part-time/full-time) and occupational prestige (manual/non-manual).
- **Working conditions** refers to the constraint level to which workers are subject and includes work schedule (atypical/regular shift), decision latitude, and latitude to organize worktime (onsite/teleworking/hybrid/flexible hours).
- **Work environment** is generally not defined by the contract but includes occupational exposure and hazards (e.g., chemical, physical), family-friendly breastfeeding workplace policies such as workplace facilities (e.g., lactation room, childcare system) and social network characteristics (e.g., parity, social support from manager or colleagues).

## Results

### Study selection

**Fig. 1:**
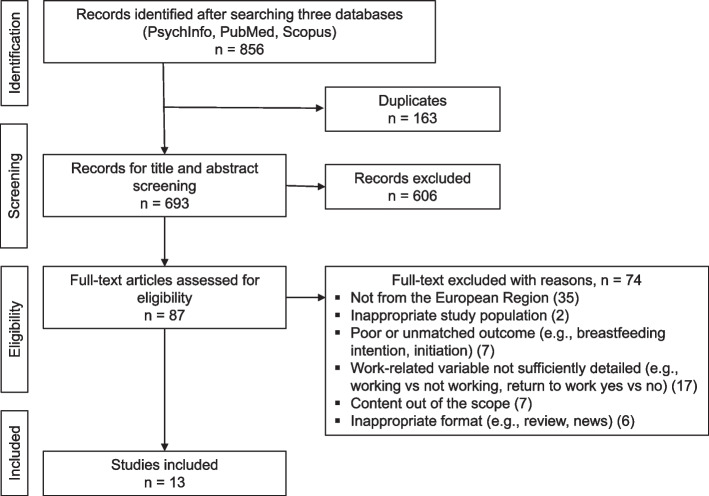
PRISMA flow diagram of study selection

A total of 856 articles were selected from the three databases (Fig. 1). After removing duplicates (*n* = 163), 693 articles were screened for possible relevance based on their title and abstract. A total of 87 studies met our inclusion criteria and were subject to a full-text review, with 13 articles meeting the eligibility criteria and being included in this scoping review.

### Characteristics of the included studies

Table 1 provides an overview of the included studies: in eight of the articles, the relation between maternal work and breastfeeding practices was main objective [18–25], while the remaining five investigated a broader spectrum of determinants [26–30]. The majority of studies were conducted in Spain (*n* = 4) and France (*n* = 4), followed by the UK (*n* = 2), Ireland (*n* = 2), and the Netherlands (*n* = 1). Eight studies were conducted on mothers sampled from the general population, whereas the others targeted mothers working at a university (*n* = 3) or immigrant mothers (Latina [*n* = 1] or Chinese [*n* = 1]). Eight studies were quantitative, and five were qualitative. There was thus substantial heterogeneity between the available studies.

**Table 1:** Characteristics of included studies (*n* = 13)

Source; location	Study design; date of the study; data collection	Study aim	Target groups n; nationality, birthplace, ethnicity, or living area	Maternal work-related variables	Analysis of work-related variables	BF outcomes^a^	Confounders	Main findings
Bonet, 2013 [21]; France	Quantitative, prospective ‘EDEN Mother–Child’ cohort; 2003–2011; interview during pregnancy, postal questionnaire at 4, 8, and 12 months	Study the associations between BF at 4 months of infant age and the sociodemographic and occupational characteristics of mothers	1,339 mothers who BF their infant at discharge from the maternity unit; French 80%	Employment and return to work at 4 months after birth: - not employed before birth - employed before birth, did not return to work - employed before birth, returned to work Return to work after birth: - return to work at 4 months or before/full-time - return to work at 4 months or before/part-time - return to work between 5 and 8 months/full-time - return to work between 5 and 8 months/part-time - no return to work at 8 months	Covariate in logistic regression (sub-sample *n* = 979)	Any BF and exclusive BF rates at 4 months	Maternal age, education, parity, matrimonial status, smoking status at 4 months after birth, return to work after birth, family origin, family income, infant sex, study center	In the adjusted model, any or exclusive BF rates did not differ between women working full-time or part-time at 12 months
Castetbon, 2020 [22]; France	Quantitative prospective, ‘Epifane’ population-based birth cohort; January-April 2012; phone interviews at 1, 4, 8, and 12 months	Study the time span during which women employed prior to pregnancy returned to work according to BF duration category and identify the sociodemographic, behavioral, work, and pregnancy characteristics of women who continued BF after returning to work	2,480 mothers who worked prior to pregnancy; French 86%	Occupational group before pregnancy: - farmers - manual workers - commercial workers - managers - intermediate professionals - manual workers	Covariate in logistic regression	Any BF duration (for subsample of 1,487 women who returned to work within a year postpartum)	Maternal age, education, parity, nationality, occupation before pregnancy, matrimonial status, body weight status before pregnancy, smoking status before pregnancy, mode of delivery, gestational age, infant birthweight	In the adjusted model, compared with managers, self-employed women (2.2 (1.1, 4.5)) were more likely to combine BF and work, while intermediate professionals (0.6 (0.4, 0.8)) or manual workers (0.5 (0.3, 0.9)) were less likely to combine BF and work
Cervera-Gasch, 2020 [18]; Spain	Quantitative, retrospective cross-sectional study; 2016; online questionnaire	Analyze the perception of support given to BF workers to continue BF at two Spanish universities and the associated factors	777 mothers who gave birth in the past 10 years and work at two public universities in Spain; Spanish 98%	Occupation: - administration, services personnel - lecturer, researcher Continued BF when back at work: - yes/no Worked a shorter working day: - yes/no Mother’s perception of their workplace BF support (four dimensions of WBSS) - break time - environmental support - technical support - workplace policy	Bivariate analysis	BF continuation after return to work	_	Factors associated with longer BF duration were: BF support policy at the university and special arrangements (*p* < 0.001); participating in BF support groups (*p* < 0.001); intending to continue BF after returning to work (*p* < 0.001); awareness of the occupational legislation in force (*p* = 0.009); and having a female supervisor (*p* = 0.04)
De Lauzon-Guillain, 2019 [19]; France	Quantitative, prospective, ‘ELFE’ longitudinal birth cohort; April 2011; interview after delivery, telephone interview at 2 and 12 months, and monthly questionnaire from 3 to 10 months	Describe the association between BF initiation and duration and the statutory duration of postnatal maternity leave, the gap between the end of legal maternity leave and the mother’s return to work, and maternal working time during the first year postpartum	8,009 mother–child pairs; French 94%	Occupational grade: - farmers, retail traders, tradeswomen - managers - intermediate professionals - employees - manual workers Type of contract: - non-permanent position - permanent position Working time: - part-time in pregnancy - full-time in pregnancy and not working at 1 year - full-time in pregnancy and part-time at 1 year - full-time in pregnancy and at 1 year	Risk factor in multinomial logistic regression	Any BF duration: - 1 to < 3 months - 3 to < 6 months - 6 to < 9 months - at least 9 months	Maternal age at first child’s birth, parity, nationality, education, pre-pregnancy BMI, smoking status, infant sex, birth weight, paternal age difference with mother, paternal presence at delivery, family type, family income, study design-related characteristics (maternal region of residence, size of maternity unit, recruitment wave)	Working part‐time at 1 year postpartum compared with full-time was positively related to BF duration, especially for primiparous women: - primiparous mothers working part-time at 1 year were more likely to BF for at least 9 months (vs BF 3–6 months: 1.8 (1.2, 2.7)) - mothers with a second child and working part-time at 1 year were less likely to BF with a shorter duration of 1 to < 3 months (vs BF 3–6 months: 0.7 (0.5, 0.9)) - mothers with three or more children and working part-time at 1 year were more likely to BF 6 to < 9 months (vs BF 3–6 months: 1.99 (1.1, 3.7))
Desmond, 2016 [20]; Ireland	Qualitative, descriptive; April-June 2014; semi-structured telephone interviews (*n* = 15) or face-to-face interview (*n* = 1)	Investigate the barriers to return to work for BF mothers and explore the experiences of mothers who continued to provide breast milk to their babies after returning to work	16 mothers who continued to BF after returning to work; Irish 94%	N.A	Thematic analysis	Any BF experience	_	Work-related barriers to BF: - lack of facilities to express and store breast milk at work - lack of time to express milk during the working day - negative attention or lack of social support (from manager and colleagues)
Hentges, 2021 [23]; Netherlands	Qualitative, descriptive; March–May 2020; semi-structured online interviews	Identify perceptions and experiences of mothers employed at Dutch universities regarding barriers and enablers to workplace BF and pumping	13 mothers who BF or pumped at work within the past 5 years and working at Dutch universities as academic staff; Dutch 85%	Occupation at the university (open-ended question) Working hours/week (open-ended question): - 16, 20, 24, 32, 40 h Type of contract: - permanent - fixed-term	Thematic analysis	Any BF duration and experience	_	No difference in BF duration between working hours (part-time vs full-time) (*p* > 0.05) Work-related barriers to BF: - inappropriate and inaccessible lactation rooms - lack of communication and information-provision - other people’s lack of awareness - inflexible working hours and unadjusted workloads, especially for teaching positions
Huet, 2016 [28]; France	Quantitative, cross-sectional observational survey; 2012; questionnaire administered by doctors during pediatric consultation	Evaluate the mean duration of exclusive BF nationally and regionally and identify its clinical and socioeconomic determinants	2,773 mothers wishing to stop exclusive BF within 7 days of consultation or having stopped within 7 days before consultation; French resident 100%	Occupational group: - farmers, retail traders, tradeswomen - managers - intermediate professionals - employees - manual workers - unemployed	Covariate in logistic regression model	Exclusive BF duration	Maternal age, smoking status, alcohol status, financial difficulties, working status, BF motivation (believed that BF was practical or favored the mother–child relationship), difficulty in starting BF, feeling uncomfortable BF in public, enjoying BF, infants’ digestive problems	Mothers were 1.8 times more likely to continue exclusive BF after 4 months when the workplace had implemented a strong BF policy (1.8 (1.1, 2.8)) compared with a minimal or moderate BF policy
Iglesias-Rosado, 2021 [29]; Spain	Qualitative, descriptive; November 2019-June 2020; semi-structured interview by video calls	Exploring Latina immigrants’ BF experiences in a Spanish-speaking country	19 Latina immigrant mothers who gave birth in Spain and experienced BF in the host country for at least 2 months over the last 5 years	N.A	Thematic analysis	Any BF experience	_	Work-related barriers to BF: - absence of adequate BF facilities
Jackson, 2021 [26]; UK	Qualitative, descriptive; April-June 2018; semi-structured face-to-face interviews (*n* = 20) or telephone interviews (*n* = 5)	Examine the motivations and experiences of British women who BF past infancy	24 mothers who BF at least one child past 12 months; British 92%	N.A	Thematic analysis	Any BF experience	_	Work-related barriers to BF: - difficulty asking for time to express in the workplace, especially in male-dominated environments - lack of facilities for expressing milk - stress caused by male gazing
Leon-Larios, 2019 [24]; Spain	Quantitative, retrospective cross-sectional study; January-March 2017; web-based questionnaire	Explore women’s experience with continuing BF when they returned to work	197 mothers employed at the University of Seville who gave birth in the last 10 years while working at university; Spanish 97.5%	Position at university: - faculty member - administrative staff Mother’s perception of their workplace BF support (four dimensions of WBSS) - break time - environmental support - technical support - workplace policy	Chi2	Any BF continuation	_	Faculty members took more breaks for BF (*p* = 0.002) and were able to organize breaks more easily (*p* < 0.001) than administrative staff Easier for faculty members to find a quiet place to pump breast milk (*p* = 0.025) Faculty members were more likely to continue BF after returning to work than administrative staff (*p* < 0.009)
Villar, 2018 [27]; Spain	Quantitative, cohort; 2003–2008; Questionnaires during the first and third trimester of pregnancy and 6 and 14 months post-delivery	Identify the factors associated with non-initiation and cessation of predominant BF	2,195 mother-infant pairs; Spanish 92%	Employment at week 32 of pregnancy and at 14 months postpartum: - working or not working Social class: - manual or non-manual occupation	Covariate in logistic analysis cox model	Predominant BF duration	Maternal age, education, parity, physical activity, weight status, smoking status in pregnancy, cohort/region	Rates of predominant BF after 13 or 16 weeks were significantly higher in non-manual working mothers (compared with manual working mothers, all *p* < 0.001) In the adjusted model, the type of work (manual vs non-manual) was not significantly associated with the likelihood of predominant BF cessation
Zhou, 2020 [30]; Ireland	Qualitative, descriptive; December 2009-February 2010; semi-structured individual interviews	Identify the factors contributing to the success of exclusive BF in Chinese immigrant mothers and find solutions to the barriers impeding exclusive BF	14 Chinese immigrant mothers who gave birth in Ireland, BF their child for at least 6 months, and exclusively BF for 4 to 6 months	Occupation (open-ended question): - stay-at-home - part-time non-professional work - self-employed - professional work	Thematic analysis	Exclusive BF experience	_	Work-related barriers to BF: - difficulty of balancing BF and employment Work-related facilitators to BF*:* - work flexibility - childcare near their workplaces - understanding of employers
Zilanawala, 2017 [25]; UK	Quantitative, observational study (The Millennium Cohort); 2000–2002; questionnaires at 9 months	Investigate the linkages between maternal nonstandard work schedules and BF initiation and duration	17,397 mothers who gave birth in the UK between 2000–2002; white 89.9%	Frequency of work schedules: - standard (Monday to Friday, 9 am-5 pm) - evenings (6 pm-10 pm) - nights (10 pm-7 am) - weekends - overnight away from home - unemployed	Explanatory variable in multinomial logistic regression models	Partial and exclusive BF duration: - 0 to < 2 months - 2 to < 4 months - at least 4 months supplementing with solids or formula before 4 months - at least 4 months supplementing with solids or formula at 4 months or later	Maternal age, ethnicity, smoking status, education, family income, psychosocial factors (childcare arrangements; maternal attachment, malaise inventory score) child sex, child age	Fully adjusted models showed no significant differences in the odds of BF duration by maternal nonstandard work schedule (i.e., working evenings, nights, or weekend shifts)

*Abbreviations*: *BF* breastfeeding, *BMI* body mass index, *WBSS* Workplace Breastfeeding Support Scale (items measured on a Likert scale from 1 to 7 [1: strongly disagree, 7: strongly agree])

^a^Any breastfeeding: intake of breast milk and/or formula; exclusive breastfeeding: intake of breast milk only; predominant breastfeeding: intake of breast milk plus liquids like juice or water; partial breastfeeding: intake of breast milk and formula; breastfeeding: term used in the study without further clarification

### Factors related to the type of employment

A previous study revealed that compared with managers, self-employed mothers were twice as likely to combine breastfeeding and work (OR 95% CI 2.2 (1.1, 4.5)), while intermediate professionals (OR 95% CI 0.6 (0.4, 0.8)) and manual workers (OR 95% CI 0.5 (0.3, 0.9)) were less likely to combine breastfeeding and work [22]. Accordingly, Villar et al. observed higher rates of predominant breastfeeding at 13 or 16 weeks in non-manual working mothers (59 and 52%, respectively) compared with their manual counterparts (48 and 41%, respectively). However, the likelihood of breastfeeding cessation did not differ between manual and non-manual workers in the fully adjusted model (not adjusted for child’s age) [27]. Inconsistent results were found concerning the association between working time and breastfeeding. Data from a French birth cohort revealed that working part-time during the first year postpartum was associated with longer breastfeeding duration [19]. This was especially true for primiparous mothers who were more likely to breastfeed for at least 9 months compared with an intermediate duration of 3 to < 6 months when they shifted from full-time work during pregnancy to part-time work in the first year postpartum (OR 95% CI 1.8 (1.2, 2.7)). However, other studies did not observe significant differences in breastfeeding duration [23, 24] or breastfeeding rate at 4 months [21] depending on the work schedules (part-time vs full-time).

### Factors related to the work conditions

Zilanawala et al. investigated maternal nonstandard work schedules and breastfeeding duration: no differences in the odds of breastfeeding duration patterns (i.e., less than 2 months, between 2 and 4 months, more than 4 months) were shown in terms of mothers’ nonstandard working schedules (i.e., working evenings, nights, or weekend shifts) in the fully adjusted models [25]. Lack of time or flexibility to express milk at work was cited by mothers as a barrier to breastfeeding in several qualitative studies [20, 23, 30] but also discussed as a potential explanatory factor of deleterious breastfeeding outcomes in other studies, which nevertheless did not measure lack of time or flexibility [18, 19, 21, 22]. Only two studies [18, 24] targeting Spanish mothers working at universities have quantitatively measured the ‘Break Time’ dimension using the Workplace Breastfeeding Support Scale (WBSS) [31]. This dimension measures, for example, mothers’ perception of the frequency and duration of their break time (e.g., “My breaks are frequent/long enough for breastfeeding or pumping breast milk”) but also their time flexibility (“I can adjust my break schedule in order to breastfeed or pump breast milk”) on a 7-point Likert scale. Both studies showed that compared with administrative staff, faculty members took more breastfeeding breaks and were able to organize their breaks more easily. Faculty members were also more likely to continue breastfeeding after returning to work [24]. However, in these studies, the ‘Break Time’ dimension was not assessed according to breastfeeding outcomes.

### Factors related to the work environment

Working environment factors were systematically highlighted in qualitative studies exploring nursing mothers’ experiences [20, 23, 26, 29, 30]. The cited breastfeeding facilitators were mostly related to the possibility and ease for mothers to express milk during working hours: availability of adequate breastfeeding facilities (i.e., quiet lactation room with cleaning and storage facilities) [18, 23, 29] or the existence of childcare near the workplaces [30]. In their quantitative study, Leon-Larios et al. showed that compared with administrative staff, faculty members had easier access to quiet places to pump breast milk and breastfed for longer (association between access to pumping room and breastfeeding duration not assessed) [24]. Broadly, the workplace breastfeeding policy seems to play a major role: as reported by a French study, women were more likely to breastfeed for more than 4 months when their workplace had implemented a breastfeeding-friendly policy (OR 95% CI 1.8 (1.1, 2.8)), fully adjusted model) compared with those which did not [28]. When comparing breastfeeding duration between two universities with contrasting breastfeeding policies, Cervera-Gasch et al. highlighted that the factors associated with longer breastfeeding were the university having a breastfeeding support policy and special breastfeeding facilities; participating in breastfeeding support groups; intending to continue breastfeeding after returning to work; knowing the occupational legislation in force; and having a female supervisor [18]. In line with the latter, the negative attitude of managers and colleagues, the perceived lack of support from them, the difficulty of asking for time to express in the workplace, especially in male-dominated environments, and the stress caused by male gazing were all breastfeeding barriers identified by working mothers [20, 26].

## Discussion

**Fig. 2:**
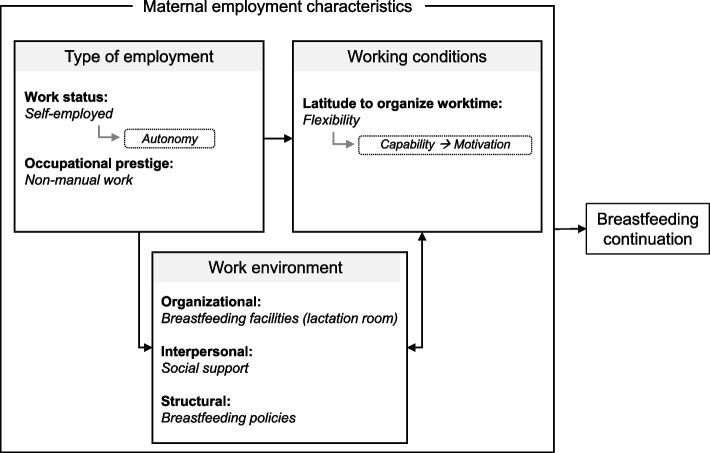
Maternal employment characteristics that support any breastfeeding continuation when returning to work in European countries. Maternal employment characteristics were grouped into three main dimensions. The type of employment dimension refers to the terms that govern the organization of work, generally stated in the contract between the employer and employee. The working conditions dimension refers to the level of constraints to which workers are subjected. The work environment dimension pertains to factors generally not defined by the contract (e.g., family-friendly breastfeeding workplace policies, occupational exposure, social network)

This scoping review aimed to identify maternal employment characteristics that support any breastfeeding continuation when returning to work in the WHO European Region. To better highlight the characteristics of employment that can lead to social inequalities, we proposed a classification through three main dimensions: type of employment, working conditions, and work environment. While these dimensions are interrelated, our review highlights that no study to date has combined all three dimensions in their measured variables. Furthermore, there is a large heterogeneity of measured work-related and breastfeeding variables, time frames, and fields of inquiry, thus revealing the lack of a conceptual framework for the links between work, breastfeeding, and social health inequalities. Nevertheless, it appears that being self-employed or working in a non-manual occupation with time flexibility, the availability of breastfeeding facilities at work, the support of co-workers, and the existence of a breastfeeding workplace policy are salient factors that promote breastfeeding among working mothers. These results are interpreted in Fig. 2.

Being self-employed implies a high level of autonomy with an early return to work. This work status was associated with longer breastfeeding duration in France [22]. As emphasized by authors, the autonomy inherent in the self-employed status can be seen as a factor favoring flexibility and thus the continuation of breastfeeding. Nevertheless, it also implies a greater dedication to work and less institutional support, which would affect the initiation of breastfeeding. A longitudinal Australian cohort study illustrated this duality by showing that women in occupations with higher levels of autonomy and limited hazards (e.g., exposure to extreme noise, temperature levels, chemicals) were more likely to intend to breastfeed and initiate it [32]. Unfortunately, in the articles identified by this scoping review, occupational exposure was neither measured nor investigated. Finally, it appears that non-manual jobs positively influence breastfeeding [28, 33]. These working mothers from socially advantaged backgrounds and with higher education levels probably have a higher degree of health awareness, better health literacy, greater autonomy over their work schedule, more resources to seek help, and better compliance with the existing recommendations [33, 34]. In agreement, breastfeeding surveys conducted in 19 European countries showed that a low education level is associated with a lower initiation of breastfeeding and earlier weaning [35]. As underlined by several European studies, the promotion, protection, and support of breastfeeding should be provided to all breastfeeding mothers, with specific interventions tailored to the more disadvantaged groups such as young and less educated mothers [23, 33, 35, 36]. Alternative explanations could be that manual working mothers are more likely to stop breastfeeding when resuming work than their non-manual counterparts, so as not to add to the stress or fatigue of their already physically demanding job. As stressed by Rollins et al., the impact of work on breastfeeding is multidimensional, including fatigue and practicality [5]. A French survey conducted on 1,000 women showed that breast pain, fatigue, and back pain were the main difficulties encountered during breastfeeding [37].

In terms of the work conditions dimension, the qualitative studies show that worktime flexibility is a major facilitator of breastfeeding continuation. Having the freedom to organize their own working time can potentially increase breastfeeding mothers’ capability, which refers to whether people have the knowledge, skills, and abilities required to engage in a particular behavior. Based on the framework of behavior change by Michie et al., capability influences motivation, which plays a major role in breastfeeding practices [38–40]. A recent Spanish study showed, for example, that women who decided to opt for exclusive breastfeeding and maintain it “as long as I can” were five times more likely to meet their expectations than women who set less ambitious expectations concerning exclusive breastfeeding duration [41]. Overall, these results highlight that employment may influence the entire breastfeeding process from intention to continuation. Indeed, breastfeeding intention – which is the strongest predictor of breastfeeding initiation and duration – is formed during pregnancy [39, 42]. The mother’s choice could be influenced by the anticipation of their expected work-life balance after resuming work [43, 44].

Regarding the work environment dimension, the studies summarized here identified a key feature, namely the importance of a set of underlying conditions: organizational (i.e., presence of adequate lactation room, childcare close to the workplace), structural (i.e., breastfeeding policies in the workplace), and even interpersonal conditions (i.e., support from co-workers), which must coexist to allow mothers to express their milk. In line with the interpersonal dimension, it was underlined that female-dominated environments were perceived to be more positive and supportive, thus enhancing breastfeeding practices [18, 23, 24]. A female environment would facilitate communication and shared experiences [23]. Findings from a study in the US showed that compared with female coworkers, males were more stigmatizing to lactating colleagues, had more responses of disgust, had a poorer perception of the fairness of the additional break time accorded for pumping breast milk, and showed less support [45]. Recent literature reviews and meta-analysis unanimously pointed out the lack of research on the effectiveness of interventions to support breastfeeding in the workplace in high-income countries, specifically in the European Region [46–49]. As demonstrated in 2022 by Tomori et al. in their review of reviews, inadequate attention is given to interventions addressing policy and structural factors, and only 9% addressed workplace intervention settings [48].

Our results should also be considered according to different national parental leave and return-to-work policies that play a crucial role in influencing breastfeeding practices. The five countries represented in our corpus (i.e., Spain, France, UK, Ireland and the Netherland) have different statutory well-paid maternity leaves durations that vary from 16 weeks (Spain, France and the Netherland) to 39 weeks (UK) whilst paternity leaves durations vary from 1 week (UK and The Netherland) to 16 weeks (Spain) [50]. Additional parental leaves are generally low or unpaid, inflexible, and not evenly distributed between fathers and mothers, because of the conservative division of gender roles predominant in these countries [4, 51]. Conversely, Sweden, which has one of the most generous, supportive and equitable parental leave programs in the world provides some insights into the integration of breastfeeding and women’s employment [52]. A cross sectional study among Swedish families revealed that a longer period of shared parental leave was associated with an extended duration of breastfeeding [53]. Thus, from national policy directives to sociocultural attitudes and values, maternal employment conditions play a crucial role to improve breastfeeding.

This study has several limitations. Inherent to the design of scoping reviews, we did not assess the methodological quality of the included papers, and so we only discuss general, albeit, limited findings regarding breastfeeding and maternal employment. This work lacks representativeness, since only five of the 53 countries included in the WHO European Region were represented in our study selection with an exclusive representation of the countries in North-West and Southern Europe. Finally, from a methodological point of view, we observed heterogeneity in the description and analysis of maternal work-related variables, thus making comparisons difficult across studies. As underlined by some authors, data on work characteristics were often limited [22], and job title classifications should be homogenized throughout the European Region [54]. While not investigated in our corpus, we may assume that other stressor factors such as job insecurity, occupational exposure to chemicals, and physical strain may also affect breastfeeding practices. Given that some studies from our corpus did not specifically aim to assess the associations between breastfeeding and maternal work, the infant’s age at the time of breastfeeding cessation was not always reported or considered in the adjusted models: this made it difficult to interpret the reason for breastfeeding cessation (e.g., work-related, meeting expectations, duration regarded as sufficient). The strength of this scoping review lies in its innovative approach by considering maternal employment characteristics in light of social inequalities. Broadly, and as conceptualized by the WHO [15], employment conditions can lead to social health inequalities through numerous behavioral, psychosocial, and physio-pathological pathways: employment conditions (e.g., full-time work, precarious employment) influence working conditions (e.g., physical and chemical hazards, ergonomics, psychosocial), and both are affected by social and family networks, health system, material deprivation, and economic inequalities. The scoping review methodology allowed us to apply a broad research question and iterative search strategy to gain a comprehensive overview of the current literature on maternal work characteristics and breastfeeding as a major public health outcome. Additionally, we considered the association between maternal work characteristics and any types of breastfeeding, without restricting the analysis to exclusive breastfeeding. We believe that this inclusive approach is relevant given the beneficial effects of breastfeeding, even partial, compared with not breastfeeding [55, 56].

## Conclusions

This review highlights that the pursuit of breastfeeding after returning to work is associated with various work characteristics that act at different interrelated dimensions (i.e., type of employment, working conditions, and work environment). Supporting disadvantaged working mothers who choose to breastfeed is all the more important given the myriad of adverse factors to which underprivileged mother and child dyads are exposed. Results from our review suggest the need for policy directives or workplace interventions to improve employment quality in order to favor work-life balance: targeting low skilled or precarious jobs by increasing flexibility and reorganizing manual work posts to be less stressful could be a relevant perspective to reduce social health inequalities broadly, and in particular, in relation to breastfeeding practices. Widely, promoting work-life balance at this crucial moment of child arrival must address the issue of gender inequalities in domestic labor. This work also advocates for actions at a more macroscopic level with the implementation of well-paid, flexible and equitable parental leave regulations between both parents in Europe. From a methodological perspective, there is an additional need for a rigorous and homogenous assessment of maternal employment characteristics in studies in order to better understand the specificities that mothers face in the workplace – including potential stressors like job insecurity, occupational exposure to chemicals, or physical strain – and to identify targeted actions. Furthermore, better quantifying worktime flexibility in studies could be of interest, since this aspect seems to play a major role in the pursuit of breastfeeding after returning to work. The new working practices adopted since the COVID-19 pandemic have challenged this link between work-life balance and social health inequalities, since precarious employees, including manual workers, are less likely to work from home.

### Supplementary Information

Additional file 1. Macro-theoretical framework of employment relations and health inequalities from the WHO Commission on Social Determinants of Health (CSDH) Employment Conditions Knowledge Network (EMCONET), Final Report, 20 September 2007.

Additional file 2. Literature Search Strategy.

## Data Availability

All data generated or analysed during this study are included in this published article [and its supplementary information files].
