# Supplementary material for: Maternal employment characteristics as a structural social determinant of breastfeeding after return to work in the European Region: a scoping review
Source: Int Breastfeed J. 2024 May 28;19:38. doi: 10.1186/s13006-024-00643-y (PMC11134638; doi:10.1186/s13006-024-00643-y)
Supplement: Supplementary file 1 — Additional file 1. Macro-theoretical framework of employment relations and health inequalities from the WHO Commission on Social Determinants of Health (CSDH) Employment Conditions Knowledge Network (EMCONET), Final Report, 20 September 2007. [file 13006_2024_643_MOESM1_ESM.docx]

**Additional file 1**: Macro-theoretical framework of employment relations and health inequalities from the WHO Commission on Social Determinants of Health (CSDH) Employment Conditions Knowledge Network (EMCONET), Final Report, 20 September 2007.


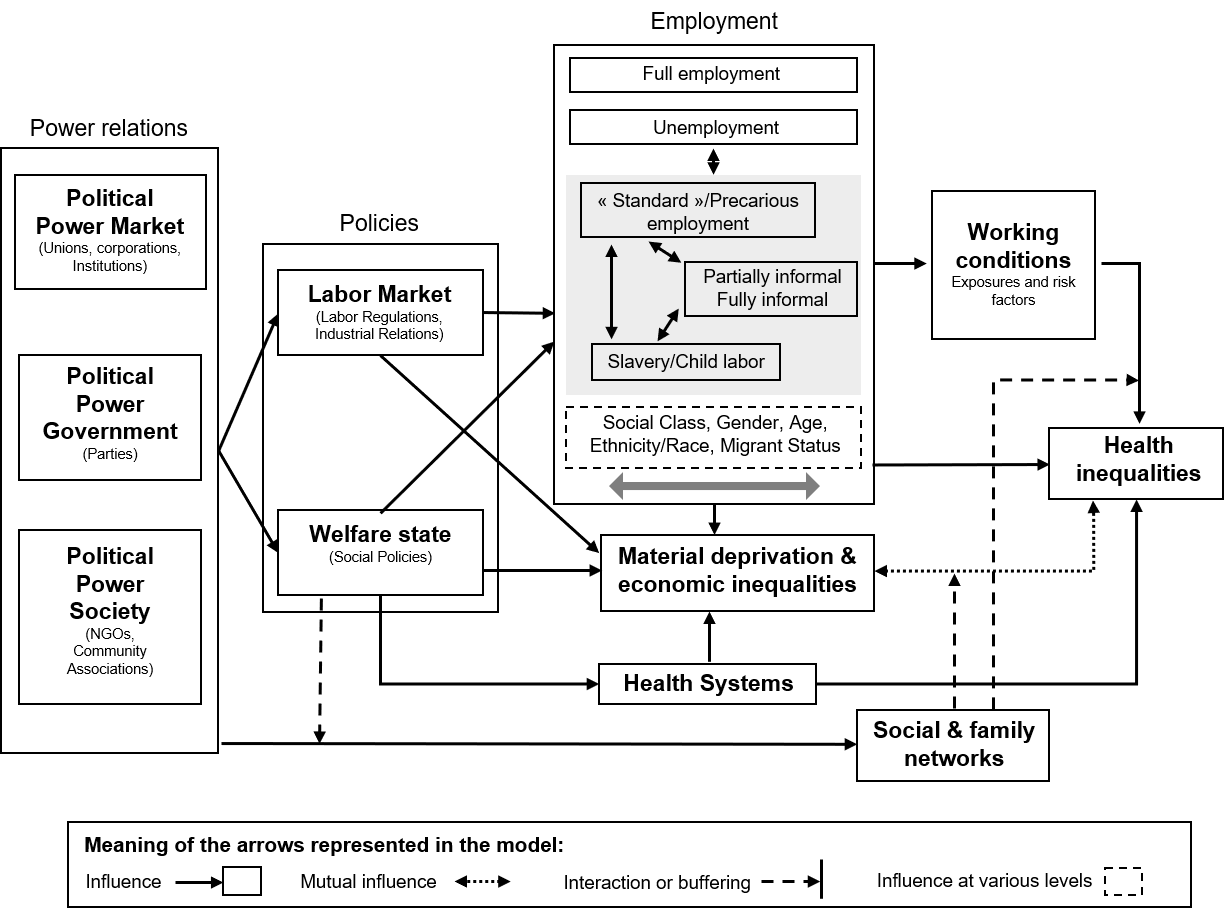


Source: Benach, J., Muntaner, C., & Santana, V. (2007). Employment conditions and health inequalities.
