# Supplementary material for: Maternal employment characteristics as a structural social determinant of breastfeeding after return to work in the European Region: a scoping review
Source: Int Breastfeed J. 2024 May 28;19:38. doi: 10.1186/s13006-024-00643-y (PMC11134638; doi:10.1186/s13006-024-00643-y)
Supplement: Supplementary file 2 — Additional file 2. Literature Search Strategy. [file 13006_2024_643_MOESM2_ESM.docx]

**Additional file 2**: Literature Search Strategy

Full electronic search strategy in Scopus, PubMed and PsychInfo databases performed on October 22, 2022 and updated on March 20, 2023. Restrict to articles published in English or French in the past 10 years (2013-2023).

*Scopus*

TITLE(Breastf* OR "Breast F*" OR (mother* W/3 milk) OR "Infant Feeding") AND (TITLE-ABS-KEY(((*employ* OR work* OR occupation* OR Job) W/3 (mother OR maternal OR women)) OR "work related" OR "Occupation* related" OR Workplace OR ((parental OR matern* OR Mother OR Breastf* OR "Breast F*") W/3 leave) OR ((job OR Work* OR *employ* OR Occupation*) W/3 (characteristic OR Status OR condition OR Schedule)) OR Shift-work* OR Shiftwork OR "return* to work" OR self-employed) OR KEY("Women Working"))

*PubMed*

(Breastf*[TI] OR "Breast F*"[TI] OR "mother’s milk"[TI] "Mothers’ own milk"[TI] OR "Infant Feeding"[TI] OR "Breast Feeding"[MAJR]) AND ("mothers employed"[TIAB] OR "working mother*"[TIAB] OR "mother's occupation*"[TIAB] OR "Mothers work*"[TIAB] OR "employed mother*"[TIAB] OR "employed women"[TIAB] OR "mother’s employ*"[TIAB] OR "mother's job*"[TIAB] OR "maternal employ*"[TIAB] OR "maternal work*"[TIAB] OR "unemployed mother*"[TIAB] OR "unemployed women"[TIAB] OR "women's employment"[TIAB] OR "self employed"[TIAB] OR "worked part time"[TIAB] OR "Working part-time"[TIAB] OR "Work part-time" [TIAB] OR "worked part time"[TIAB] OR "part time work*"[TIAB] OR "part-time employed"[TIAB] OR "employed part-time"[TIAB] OR "full-time work*"[TIAB] OR "working full-time"[TIAB] OR "work full-time"[TIAB] OR "full-time employ*"[TIAB] OR "full time job"[TIAB] OR "employed full time"[TIAB] OR "informally working"[TIAB] OR "Informal work"[TIAB] OR "Informal occupation*"[TIAB] OR "informally employ*"[TIAB] OR "shift work*"[TIAB] OR Shiftwork*[TIAB] OR "returning to work"[TIAB] OR "Return to work" [TIAB] OR "characteristics of job*"[TIAB] OR "Job characteristic*"[TIAB] OR "employment characteristic*"[TIAB] OR "occupational characteristic*"[TIAB] OR "work characteristic*"[TIAB] OR "working characteristic*"[TIAB] OR "employment status"[TIAB] OR "working status"[TIAB] OR "work status"[TIAB] OR "occupational status"[TIAB] OR "working condition*"[TIAB] OR "work condition*"[TIAB] OR "employment condition*"[TIAB] OR "Work related"[TIAB] OR "Occupation related"[TIAB] OR Workplace*[TIAB] OR "Maternity leave*"[TIAB] OR "Parental leave*"[TIAB] OR "breastfeeding leave*"[TIAB] OR "paternity leave*"[TIAB] OR "Parental Leave"[Mesh] OR "Women, Working"[Mesh] OR "Workplace"[Mesh] OR "Return to Work"[Mesh])

*PsychInfo*

#1: TI (Breastf* OR "Breast F*" OR "mother’s milk" "Mothers’ own milk" OR "Infant Feeding") OR MJ "Breast Feeding"

#2: TI ("mothers employed" OR "working mother*" OR "mother's occupation*" OR "Mothers work*" OR "employed mother*" OR "employed women" OR "mother’s employ*" OR "mother's job*" OR "maternal employ*" OR "maternal work*" OR "unemployed mother*" OR "unemployed women" OR "women's employment" OR "self employed" OR "worked part time" OR "Working part-time" OR "Work part-time" OR "worked part time" OR "part time work*" OR "part-time employed" OR "employed part-time" OR "full-time work*" OR "working full-time" OR "work full-time" OR "full-time employ*" OR "full time job" OR "employed full time" OR "informally working" OR "Informal work" OR "Informal occupation*" OR "informally employ*" OR "shift work*" OR Shiftwork* OR "returning to work" OR "Return to work" OR "characteristics of job*" OR "Job characteristic*" OR "employment characteristic*" OR "occupational characteristic*" OR "work characteristic*" OR "working characteristic*" OR "employment status" OR "working status" OR "work status" OR "occupational status" OR "working condition*" OR "work condition*" OR "employment condition*" OR "Work related" OR "Occupation related" OR Workplace* OR "Maternity leave*" OR "Parental leave*" OR "breastfeeding leave*" OR "paternity leave*") OR AB ("mothers employed" OR "working mother*" OR "mother's occupation*" OR "Mothers work*" OR "employed mother*" OR "employed women" OR "mother’s employ*" OR "mother's job*" OR "maternal employ*" OR "maternal work*" OR "unemployed mother*" OR "unemployed women" OR "women's employment" OR "self employed" OR "worked part time" OR "Working part-time" OR "Work part-time" OR "worked part time" OR "part time work*" OR "part-time employed" OR "employed part-time" OR "full-time work*" OR "working full-time" OR "work full-time" OR "full-time employ*" OR "full time job" OR "employed full time" OR "informally working" OR "Informal work" OR "Informal occupation*" OR "informally employ*" OR "shift work*" OR Shiftwork* OR "returning to work" OR "Return to work" OR "characteristics of job*" OR "Job characteristic*" OR "employment characteristic*" OR "occupational characteristic*" OR "work characteristic*" OR "working characteristic*" OR "employment status" OR "working status" OR "work status" OR "occupational status" OR "working condition*" OR "work condition*" OR "employment condition*" OR "Work related" OR "Occupation related" OR Workplace* OR "Maternity leave*" OR "Parental leave*" OR "breastfeeding leave*" OR "paternity leave*") OR MA ("Parental Leave" OR "Women, Working" OR "Workplace" OR "Return to Work")
